# Experimental evolution of environmental tolerance, acclimation, and physiological plasticity in a randomly fluctuating environment

**DOI:** 10.1002/evl3.306

**Published:** 2022-12-07

**Authors:** Marie Rescan, Nicolas Leurs, Daphné Grulois, Luis‐Miguel Chevin

**Affiliations:** ^1^ CEFE, CNRS, Univ Montpellier, Univ Paul Valéry Montpellier 3, EPHE, IRD Montpellier 34090 France; ^2^ Université Perpignan Via Domitia, Centre de Formation et de Recherche sur les Environnements Méditerranéens, UMR 5110 Perpignan 66860 France; ^3^ CNRS, Centre de Formation et de Recherche sur les Environnements Méditerranéens, UMR 5110 Perpignan 66860 France; ^4^ ISEM, CNRS, IRD, EPHE, Univ. Montpellier Montpellier 34095 France; ^5^ Catalan Institute for Water Research (ICRA) Scientific and Technologic Park of the University of Girona Emili Grahit 101 Girona Girona 17003 Spain

**Keywords:** Acclimation, environmental autocorrelation, environmental tolerance, experimental evolution, phenotypic plasticity

## Abstract

Environmental tolerance curves, representing absolute fitness against the environment, are an empirical assessment of the fundamental niche, and emerge from the phenotypic plasticity of underlying phenotypic traits. Dynamic plastic responses of these traits can lead to acclimation effects, whereby recent past environments impact current fitness. Theory predicts that higher levels of phenotypic plasticity should evolve in environments that fluctuate more predictably, but there have been few experimental tests of these predictions. Specifically, we still lack experimental evidence for the evolution of acclimation effects in response to environmental predictability. Here, we exposed 25 genetically diverse populations of the halotolerant microalgae *Dunaliella salina* to different constant salinities, or to randomly fluctuating salinities, for over 200 generations. The fluctuating treatments differed in their autocorrelation, which determines the similarity of subsequent values, and thus environmental predictability. We then measured acclimated tolerance surfaces, mapping population growth rate against past (acclimation) and current (assay) environments. We found that experimental mean and variance in salinity caused the evolution of niche position (optimal salinity) and breadth, with respect to not only current but also past (acclimation) salinity. We also detected weak but significant evidence for evolutionary changes in response to environmental predictability, with higher predictability leading notably to lower optimal salinities and stronger acclimation effect of past environment on current fitness. We further showed that these responses are related to the evolution of plasticity for intracellular glycerol, the major osmoregulatory mechanism in this species. However, the direction of plasticity evolution did not match simple theoretical predictions. Our results underline the need for a more explicit consideration of the dynamics of environmental tolerance and its underlying plastic traits to reach a better understanding of ecology and evolution in fluctuating environments.

The range of abiotic environments over which a species can sustain positive population growth when starting at low density defines its fundamental niche (Hutchinson 1957; Holt 2009). Slices of this fundamental niche along specific abiotic axes, such as temperature or salinity, can be measured using environmental tolerance curves, which relate absolute fitness (or performance as a surrogate) to the environment (Angilletta 2009; Buckley and Kingsolver 2021; Cifuentes et al. 2001; Lynch and Gabriel 1987). As fitness and performance are highly integrated traits that depend on many upstream biological processes, environmental tolerance curves must emerge from the phenotypic plasticity of traits underlying adaptation to the environment, such that evolution of plasticity and of environmental tolerance are tightly connected (Chevin et al. 2010; Lande 2014).

Both theory and experiments have investigated the evolution of plasticity and environmental tolerance in response to patterns of environmental variation. Evolutionary theory has consistently shown that higher phenotypic plasticity and broader tolerance curves should evolve in environments that vary more predictably, such that the environment that elicits the plastic response is highly correlated with the environment where selection occurs (De Jong 1999; Gavrilets and Scheiner 1993; King and Hadfield 2019; Lande 2009; Levins 1963; Moran 1992). For an environment that undergoes stationary fluctuations, the expected equilibrium plasticity should thus depend on the temporal autocorrelation of the environment, which determines environmental predictability (Gavrilets and Scheiner 1993; Lande 2009). But what has been perhaps little appreciated is that these predictions hold only for a specific model of “static” or “fixed” plasticity, where the phenotype is determined once during developing, and later exposed to selection. Fewer models have considered the evolution of dynamic, labile traits that can change reversibly over time (Beaman et al. 2016). Lande (2014) showed that for a labile character that can change continuously and at constant rate over lifetime, plasticity and tolerance breadth at evolutionary equilibrium no longer depend on environmental predictability, in the absence of cost of plasticity. However, with a cost of plasticity (DeWitt et al. 1998; Van Tienderen 1991), the equilibrium plasticity of a labile trait increases with temporal autocorrelation of the environment (as for a fixed trait), but to a level that also depends on the magnitude of environmental fluctuations. Using another type of model, Gabriel et al. (2005) found that with reversible plasticity, the equilibrium tolerance breadth is larger when organisms have imperfect information about selection, making it less predictable. These somewhat discordant predictions about the role of environmental predictability on the evolution of plasticity and tolerance breadth probably arise from the specificities of each model, highlighting that much is left to understand about the evolution of dynamic (i.e., nonfixed) plasticity and tolerance breadth (see also Fischer et al. 2014; Ratikainen and Kokko 2019; for the interplay of plasticity with age).

On the empirical side, evolution of plasticity and environmental tolerance has been investigated in a number of experimental evolution studies (Bennett and Lenski 1997; Berger et al. 2014; Huang and Agrawal 2016; Koch and Guillaume 2020; Reboud and Bell 1997; Scheiner 2002; Thuy et al. 2016), and in the wild, where higher plasticity levels have been linked to more variable environments (Schaum et al. 2013). Only few of these studies have addressed the role of environmental predictability (however, see Dey et al. 2016; Leung et al. 2020; Mitchell et al. 2009; Scheiner and Yampolsky 1998), and the dynamic aspects of plasticity were often neglected. Leung et al. (2020) recently demonstrated that morphological plasticity evolved in response to environmental predictability, in lines of the microalga *Dunaliella salina* that had experienced randomly fluctuating salinity with the same stationary distribution, but different levels of temporal autocorrelation. In addition, the direction and magnitude of evolution of plasticity was dynamical, changing over at least 10 days (and about as many generations) after exposure to a new salinity. Strong acclimation effects were also shown to exist in this species, such that population growth at a given time strongly depends on both current and past salinities, and their interaction (Rescan et al. 2020), similar to what was shown for temperature in other microorganisms (Fey et al. 2021; Kremer et al. 2018; Leroi et al. 1994). In *D. salina*, intracellular glycerol serves as an osmoprotectant that allows population growth even at salinities close to saturation (Ben‐Amotz and Avron 1973; Borowitzka et al. 2004; Chen and Jiang 2009), and was shown to explain part of the effects of salinity acclimation on growth (Rescan et al. 2020). This highlights that intracellular glycerol content is likely a major plastic trait underlying the evolution of environmental tolerance and acclimation effects in this species.

This raises the following questions: to what extent do patterns of environmental fluctuations, especially their predictability, influence evolution of the tolerance curve with respect not only to the current, but also to previous environments, through dynamic acclimation effects? And how do these responses at the level of fitness relate to evolution of plasticity for well‐characterized mechanisms underlying environmental tolerance? To answer these questions, we exposed lines of the halotolerant microalga *D. salina* to randomly fluctuating salinity with variable predictability (tuned by their temporal autocorrelation) for about 200 generations. We then measured their tolerance curves with respect to both current and previous salinity, as well as their glycerol content, the main mechanism of osmoregulation in this species. Our results highlight that important aspects of the evolution of plasticity and environmental tolerance in fluctuating environments may be missed if lagged effects of past environments are not accounted for.

## Methods

### EXPERIMENTAL EVOLUTION IN STOCHASTIC AND CONSTANT SALINITIES

We exposed 25 populations of *D. salina* to constant or fluctuating salinities for 8–12 months (with about 1 doubling per day). To maximize initial genetic standing variation, populations were initiated by mixing 50% of strain CCAP19/15 and 50% of strain CCAP19/18 from the Culture Collection of Algae and Protozoan, but amplicon sequencing revealed that strain CCAP19/18 disappeared from most populations (see Supporting Information in Rescan et al. 2020). Populations were transferred twice a week (every three to four generations in optimal conditions; see Ben‐Amotz et al. 2009; Rescan et al. 2020) by diluting 15% of the culture into 800 μL of fresh medium using a liquid‐handling robot (Biomek NXP Span‐8; Beckman Coulter), except for a 2‐week logistic break during which all fluctuating treatments were maintained at 2.4 M, and the constant treatments at their respective fixed salinities (Fig. S1A). At each transfer, the target salinity was achieved by mixing the required volumes of hypo‐ ([NaCl] = 0 M) and hyper‐ ([NaCl] = 4.8 M) saline media, after accounting for dilution of the pretransfer salinity. Populations in constant salinities were exposed to 0.8, 2.4, and 3.2 M NaCl, with two replicates per salinity. The fluctuating treatments consisted of four or five independent stochastic salinity time series, for each of four temporal autocorrelation levels. Salinities were sampled from a first‐order autoregressive process (AR1) with stationary mean 2.4 M, variance 1, and autocorrelations ρ = –0.5, 0, 0.5, or 0.9 (where the time step for defining autocorrelation is the transfer). Parameters were chosen based on a preliminary acclimated tolerance surface, to limit extinction risk during long‐term experiment and maximize the difference in population dynamics between treatments. To keep a constant stationary variance of 1 regardless of autocorrelation, the noise term in the AR1 process was drawn from a normal distribution with variance $\left(1-\rho^{2}\right)$, as commonly done in theoretical work that aims to distinguish the influence of autocorrelation from that of the variance on adaptation to stochastic environments (Charlesworth 1993; Chevin et al. 2017; Lande and Shannon 2006; see Rescan et al. 2020 for further details on the transfers during the long‐term experiment). Due to the duration of the tolerance curve measurements, the assays were performed over 4 months. To prevent potential biases, populations from all treatments were equally distributed along days of measurement (Fig. S1A).

### ACCLIMATED TOLERANCE SURFACES

#### Data acquisition

All assays were performed in artificial saline water +2% Guillard's F/2 marine water enrichment solution (Sigma; G0154‐500ml) with controlled concentration of NaCl. Cultures (flasks and plates) were placed in a growth chamber, with temperature set at 24°C and light at 200 μmol·m^–2^·s^–1^ for 12:12 h LD cycles. For each population, 500 μL of culture was sampled from the long‐term evolution experiment. Cultures were transferred into a 15‐mL flask at [NaCl] = 2.4 M (average salinity for the fluctuating treatments) and grown for 10 days in similar light and temperature conditions. This preacclimation step was implemented both to reach population sizes large enough for the experiment, and to erase potential nongenetic effects of the last salinity experienced in the long‐term evolutionary experiment (Rescan et al. 2020).

After this preacclimation step, each population was acclimated for 7 days in 50‐mL flasks with 20 mL of saline medium at nine concentrations *S*_0_ (NaCl: 0.1, 0.5, 1.1, 1.8, 2.4, 3, 3.7, 4.3, and 4.7 M), which constitute the past or acclimation environment for the tolerance surfaces. Then after 7 days, each acclimation culture was cross‐transferred into salinities *S*_1_, which constitute the current or assay salinities for tolerance surfaces, with the same nine values as *S*_0_ (except for transfers from 0.1 to 3.7, 4.3, and 4.7 M, and from 4.7 to 0.1, 0.5, and 1.1 M, that could not be reached with the dilution level we used). Overall, this resulted in 75 different cross‐salinity transfers *S*_0_ × *S*_1_ per sample. For each population, three replicates of each of these cross‐salinity treatments were distributed across four deep‐well plates (volume *V*_total_ = 800 μL). Population density was first measured in the acclimation flasks, and the initial volume used to inoculate the assay *V*_culture_ was computed so as to reach an initial population density *N*_0_ of 5 × 10^–3^ cells·mL^−1^. Appropriate volume of a hyposaline (salinity $S_{\mathrm{hypo}}=0\;M)$ and hypersaline (salinity $S_{\mathrm{hyper}}=4.8\;M)$ solutions were then added to adjust to the target salinity *S*_1_:

$$\begin{array}{c} \;V_{\mathrm{culture}}=N_{0}\times V_{\mathrm{total}}/N_{-1},\\ \;V_{\mathrm{hyper}}=\left(S_{1}\times V_{\mathrm{total}}-S_{0}\times V_{\mathrm{culture}}\right)/S_{\mathrm{hyper}},\\ \;V_{\mathrm{hypo}}=V_{\mathrm{total}}-V_{\mathrm{culture}}-V_{\mathrm{hyper}}. \end{array}$$

All transfers were performed using a liquid‐handling robot (Biomek NXP Span‐8; Beckman Coulter), insuring a high level of repeatability. Plates were covered with plastic lids, sealed with Parafilm, and placed for 3 days in a growth chamber with same light and temperature conditions as in the evolutionary experiment. To measure the initial population density following the transfer, two extra replicates per acclimation flask were transferred into a salinity 0.36 M, known to not cause direct cell mortality in the *Dunaliella* strains used here (Leung et al. 2020; Rescan et al. 2020), and following the same protocol as for other replicates. Initial density *N*_0_ was then measured directly after transfer to these 2 × 9 wells. The standard error of initial population sizes was acceptable ($N_{0}=3911\pm 283$, coefficient of variation CV = 0.07), so for each acclimation salinity *S*_0_, we used the mean from both measures as estimator of the initial population size to analyze growth during the assay. After 3 days, population density in each assay (*N*_1_, 75 × 3 measures) was measured.

Populations sizes before (*N*_0_) and after growth (*N*_1_) were assayed by flow cytometry (Guava EasyCyte^®^). Cells were counted in a 20‐μL sample of each well (2.5% of the total culture volume). *Dunaliella* cells were isolated from debris using forward scatter (FSC), side scatter (SSC), and red (695/50 nm) and yellow (583/26 nm) fluorescence (excitation 532 nm). Flow cytometry additionally allowed us to detect recently died (or dying) *Dunaliella* cells, with similar shape, size, and yellow fluorescence as living cells, but a reduced level of red fluorescence due to chlorophyll degradation. These cells coincide with cells marked with the nucleic acid stain SytoxGreen, which only permeates dead cells. However, flow cytometry cannot detect rapidly degraded cells, for instance those that undergo programmed cell death, as documented for *D. salina* (Orellana et al. 2013; Leung et al. 2022) and other phytoplankton (Bidle 2016), such that an important fraction of deaths cells may escape detection. The number of apparent death cells is nevertheless a proxy or total deaths, so we estimated initial and final concentrations of dead cells in the wells to infer an apparent death ratio.

#### Tolerance surface analysis

Initial population size in our assays (∼5 × 10^3^ cells·mL^−1^) was much below the carrying capacity of about 1 × 10^6^ cells·mL^−1^ in *D. salina* (Rescan et al. 2020), limiting density‐dependent effects. We therefore assumed exponential growth during the 3‐day assays, to compute a net per‐capita growth rate per day. For each population *i* and salinity transfer *j* (corresponding to an interaction between acclimation salinity *S*_0_ and assay salinity *S*_1_), we thus had

(1) $$\;N_{i,j,t}=N_{i,j,0}\;\times \exp\left[R_{i,j}\times t\right],$$

where $N_{i,j,0}$ and $N_{i,j,t}$ are the initial and final densities, respectively, $R_{i,j}$ is the exponential growth rate, and *t* = 3 days is the length of the assay. Note that because of our experimental design, $N_{i,j,0}$ is the same for all populations starting from the same acclimation salinity *S*_0_. Population sizes of *Dunaliella* measured by flow cytometry followed a negative binomial distribution (Leung et al. 2020).

In a second step, we partitioned the population growth rate into an apparent growth rate *r* and an apparent mortality ratio *d*. We have previously detected a drop in the number of living cells 4 hours after a transfer from low to high salinity, implying that mortality occurs before any doublings (fig. 3c in Leung et al. 2020). We therefore assumed that the measured number of dead cells after 3 days was a fraction ($d_{i,j}$) of the initial living cells $N_{i,j,0}$, and estimated an instantaneous mortality ratio $d_{i,j}$ such that

$$\;N_{i,j,t}=N_{i,j,0}\;\left(1-d_{i,j}\right)\times \exp\left[r_{i,j}\times t\right],$$

(2) $$D_{i,j,t}=D_{i,j,0}+d_{i,j}N_{i,j,0},$$

where $D_{i,j,0}$ and $D_{i,j,t}\;$are the initial and final numbers of dead cells, respectively, measured 1–2 hours $\left(D_{i,j,0}\right)$ after transfer to 0.36 M versus 3 days $\left(D_{i,j,t}\right)$ after transfer to the new salinity. Similar to overdispersion in the number of living cells leading us to use a negative binomial rather than a Poisson distribution in equation (1), the proportion of dead cells was also highly overdispersed. We thus used beta‐binomial regressions to estimates effects on mortality, corresponding to binomial regressions (as in a typical GLM), but where the probability *d* to die after transfer is itself distributed according to a beta distribution. Apparent death ratio and growth rates were estimated for each population and each cross‐salinity transfer using the R package glmmTMB (Brooks et al. 2017).

#### Bivariate tolerance curves

We wished to fit acclimated tolerance surfaces, that is, bivariate tolerance curves with a fitness component as a function of both acclimation and assay salinities. For apparent growth rate $r_{i,j}$ for population *i* and salinity transfer *j*, we used a bivariate second‐order polynomial parameterized as

(3) $$\begin{array}{ccc} \;r_{i,j} & = & r_{i,\max}-\frac{{\left(\;S_{j,1}-\mu_{i,1}\right)}^{2}}{\sigma_{i,1}^{2}}-\frac{{\left(\;S_{j,0}-\mu_{i,0}\right)}^{2}}{\sigma_{i,0}^{2}}\\ & & +\;2k_{i}\frac{\left(\;S_{j,1}-\mu_{i,1}\right)\left(\;S_{j,0}-\mu_{i,0}\right)}{\sigma_{i,1}^{2}}, \end{array}$$

where *r*_max_ is the maximal apparent growth rate across salinities, *μ*_1_ the optimal assay salinity, and *σ*_1_ the salinity tolerance breadth in the assay environment. Similarly, *μ*_0_ is the optimal salinity during the acclimation phase (in terms of its effect on apparent growth in the assay phase), and *σ*_0_ measures the tolerance breadth with respect to the acclimation salinity. Under such parametrization, *k* measures the effect of acclimation salinity on the assay salinity optimum, and thus quantifies the strength of acclimation.

Mortality occurred especially in transfers from low to high salinity, that is, at an extremum of the bivariate salinity range, so the mortality surface was monotonic. The logit is the natural link function in a (beta) binomial regression, so we modeled the logit mortality as a linear function of previous and current salinity:

(4) $$\begin{array}{ccc} \mathrm{logit}\left(d_{i,j}\left(S_{0},S_{1}\right)\right) & = & \frac{\log\left(d_{i,j}\left(S_{0},S_{1}\right)\right)}{\log\left(1-d_{i,j}\left(S_{0},S_{1}\right)\right)}\\ & = & \delta_{i}+\delta_{i,0}S_{0,j}+\delta_{i,1}S_{1,j}+\delta_{i,01}S_{0,j}S_{1,j}. \end{array}$$

Combining both estimations, the full tolerance surface for growth rate, estimated over the 3 days of the fitness assay, was then computed as $R_{i,j}=\ln\left(\frac{N_{i,j,t}}{N_{i,j,0}}\right)/t\;$, that is (from eq. 2),

(5) $$\;R_{i,j}=\frac{\ln\left(1-d_{i,j}\left(S_{0},S_{1}\right)\right)}{t}+r_{i,j}\left(S_{0},S_{1}\right).$$

To estimate simultaneously the binomial mortality and the growth term, acclimated tolerances surfaces were fitted using the R package TMB, where the likelihood function is written by the user in C++, and maximized in R using Laplace approximation (Kristensen et al. 2015).

#### Effects of experimental evolution treatments on tolerance surfaces

To quantify the effects of patterns of salinity fluctuations on evolution of environmental tolerance, we assumed that each parameter of the tolerance surface described by equations (3) and (4) depends on the mean $\bar{S_{i}}$, variance $\sigma_{i,S}^{2}$, temporal autocorrelation $\rho_{i,S}$, and predictability $\rho_{i,S}^{2}$ of the salinity treatment experienced by each population *i* during its evolution, that is,

(6) $$\theta_{i}=\theta_{\mathrm{int}}+\theta_{\mathrm{mean}}\bar{S_{i}}+\theta_{\mathrm{var}}\sigma_{i,S}^{2}+\theta_{\rho }\rho_{i,S}+\theta_{\rho^{2}}\rho_{i,S}^{2},$$

where subscript “int” stands for intercept, and θ is any parameter of the tolerance surface: $\;r_{\max}$, μ_1_ , $1/\sigma_{1}^{2}$, μ_0_ , $1/\sigma_{0}^{2},$ $1/\sigma_{0}^{2}$ *k*, δ, δ_0_, δ_1_, and δ_01_. Note that in the fluctuating treatments, salinity time series are independent realizations of a stochastic process, such that their realized moments (mean, variance, etc.) slightly differ from their target values assuming a stationary process. We thus used theses realized salinity moments (Fig. S1) to analyze the consequences of environmental mean, variance, and autocorrelation, on the evolution of acclimated tolerance curves.

In addition, we also analyzed effects of the environmental treatments for each individual cross‐salinity transfer *S*_0_ × *S*_1_, to identify salinity transfers where evolutionary responses were most salient. We thus modeled the apparent growth rate in each salinity transfer as a function of the environmental parameters of the evolution treatment:

(7) $$r_{i,j}=r_{j,\mathrm{int}}+r_{j,\mathrm{mean}}\bar{S_{i}}+r_{j,\mathrm{var}}\sigma_{i,S}^{2}+r_{j,\rho }\rho_{i,S}+r_{j,\rho^{2}}\rho_{i,S}^{2},$$

where $r_{i,\;j}$ is the growth rate of population *i* after transfer *j* from salinity *S_j,_*_0_ to *S_j_*,_1_. Similar regressions were performed for total growth *R* or for apparent death ratio d. We analyzed equation (7) in a negative binomial regression for total and apparent growth rate, and a beta binomial regression for mortality.

### EVOLUTION OF GLYCEROL DYNAMICS

Beyond measuring tolerance curves, we tracked the dynamics of glycerol, the major mechanism of osmotic regulation in *Dunaliella*, following a transfer to fresh medium at various salinities. We focused on a subset of eight out of the 19 populations evolved in stochastic environments (two for each targeted autocorrelation: −0.5, 0, 0.5, and 0.9).

#### Glycerol measures

Following the protocol used in the acclimated tolerance surface analysis, populations were first transferred with dilution 1/5 from the evolutionary experiment to 15 mL at salinity 2.4 M, and left to grow for 10 days. They were then acclimated at low (0.5 M) or high salinities (3.5 M) in 25 mL for 7 days. After acclimation, each population from each acclimation salinity was split and transferred to 20 mL fresh medium with final salinities 0.5, 2, and 3.5 M, and we measured glycerol concentration following this transfer.

We measured total and extracellular (after cell filtration) glycerol concentrations 1 hour, 8 hours, and 3 days after transfer. For each of the 144 measures (8 populations × 6 salinity transfers × 3 time points), 800 μL Free Glycerol Reagent (Sigma Aldrich) was mixed with 200‐μL culture (total glycerol concentration) or 200 μL of culture where cells had been previously removed (5 minutes, 2000 rpm on 0.2‐μm filtration plates), to measure the extracellular glycerol concentration. We measured optical density at 540 nm after 5‐minute incubation at 37°C. Glycerol concentration was interpolated from a linear standard curve (Appendix A). *Dunaliella* densities were estimated by flow cytometry to yield measurements per unit cell, and we made two independent replicates for each measure.

#### Glycerol dynamics analysis

For each population, salinity treatment, and time point, we obtained estimates and standard errors from the two replicates of cells count and total and extracellular glycerol concentrations. Subtracting the extracellular from the total glycerol concentration gave the intracellular concentration. All estimated concentrations were then divided by cell density, to obtain a scaled glycerol amount *G* per cell (mol/cell). For each data point, estimates and standard errors for these scaled metrics were computed from estimates and standard errors of glycerol and cell density by using the delta method.

To quantify the evolutionary consequences of patterns of environmental fluctuations on the dynamics of glycerol following salinity transfers, we regressed glycerol content per cell *G* against parameters of salinity fluctuations, by applying equation (6) with θ=G, in each compartment (intracellular, extracellular, or total), salinity transfer $S_{0}\times S_{1}$, and time posttransfer (1 hour, 8 hours, or 3 days). We finally performed an ANOVA to test which parameter (assay and acclimation salinities, timing of the measure, salinity mean, variance, autocorrelation and predictability during evolution, and their interactions) affected glycerol content, using the following linear model (in R formulation):

$$\begin{array}{ccc} & & \mathrm{glycerol}\left(S_{0},S_{1},\mathrm{Time}\right)\;∼\;\left(S_{0}\times S_{1}\times \mathrm{Time}\right)\\ & & \times \left(\rho_{S}^{2}+\rho_{S}+\bar{S}+\sigma_{S}^{2}\right). \end{array}$$

In particular, any significant interaction between our evolutionary treatment $\left(\rho^{2},\;\rho ,\;\bar{S},\;\mathrm{or}\;\sigma^{2}\right)$ and assay or acclimation salinity would evidence evolution of plasticity of glycerol content in response to the given parameter of the fluctuating treatment.

To propagate the error in estimates of glycerol concentrations to the parameters of their dependency on fluctuation patterns, we simulated 1000 datasets where glycerol concentrations were sampled from normal distributions parameterized by the estimates and standard errors based on true observations, and regressed glycerol level against parameters of environmental fluctuations in each dataset. We then used the R packages MICE (van Buuren and Groothuis‐Oudshoorn 2011) and MICEADDS (Robitzsch and Grund 2022) to obtain the total uncertainty, correcting for multiple imputation.

## Results

We analyzed the evolution of acclimated salinity tolerance surfaces in 25 populations of the microalga *D. salina* that had been exposed for about 200 generations to constant or stochastic salinities, with different mean, variance, and autocorrelation. We measured their per capita population growth rates, and partitioned apparent death from subsequent apparent growth, in 75 cross‐salinity treatments combining nine acclimation and nine assay salinities.

### SALINITY TOLERANCE SURFACES INVOLVE STRONG ACCLIMATION EFFECTS

**Figure 1:**
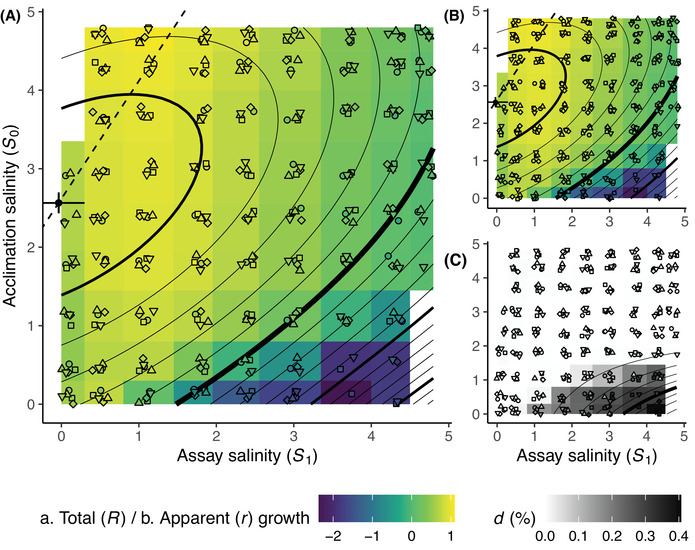
Acclimated tolerance surfaces. The rates of net growth *r* (A), apparent growth *r* (B), and apparent death *d* (C) as functions of acclimation (*S*_0_, *y*‐axis) and assay (*S*_1_, *x*‐axis) salinities are represented for the five populations that evolved under unpredictable fluctuations in salinity (white noise: mean $\bar{S}=2.4\;M$, variance $\sigma_{S}^{2}=1$, and autocorrelation $\rho_{S}=0$). Colored dots give the growth and apparent death ratios fitted independently for each population in each salinity transfer from *S*_0_ to *S*_1_. Background colors gives the mean estimates of the five populations. Contour lines are isoclines of the growth and the death function estimated by equations (3) to (5). Dots and error bars in panels (A) and (B) give the estimate and standard error for the salinity optimum. Dashed line displays current salinity optimum as a function of acclimation salinity (slope 1/*k*, see eq. 3).

*Dunaliella* had a broad niche with respect to the assay salinity *S*_1_ (Fig. 1, *x*‐axis), with a salinity optimum (μ_1_, Table 1) close to 0, and a tolerance breadth of about 4.2 M (σ_1_), allowing for positive growth at all salinities. This pattern is characteristic of a halotolerant rather than halophile species, which can deal with salinities close to saturation, but actually grows better at low salinities, including nearly fresh water. The maximal growth rate of 1.15 (Fig. 1B) corresponds to 1.66 doublings per day.

**Table 1:** Parameters of the tolerance surface in response to the acclimation and assay salinities (eqs. 3 and 4), for populations that evolved under unpredictable fluctuating salinity (white noise)

Parameter	Estimate	Standard Error
*r*_max_	1.153	0.038
k	0.666	0.068
μ_0_	2.565	0.131
μ_1_	−0.035	0.332
σ_0_	3.101	0.085
σ_1_	4.143	0.202
δ	−3.994	0.158
δ_0_	−0.033	0.072
δ_1_	1.185	0.054
δ_01_	−0.444	0.029

A striking result, consistent with previous demographic observations in this species (Rescan et al. 2020), was that the acclimation salinity *S*_0_ (*y*‐axis in Fig. 1) strongly impacted population growth during the assay. The acclimation niche (width on the *y*‐axis), capturing current tolerance to past environments, was slightly narrower (3.1 M) than the assay niche, and centered around 2.56 M, which is close to the mean salinity of the environmental treatments (2.4 M). In addition, the acclimation salinity also influenced the response to the current assay salinity, especially for acclimation salinities below *S*_0_ = 1.5 M (Fig. 1). The effect of acclimation salinity on current salinity tolerance (for apparent and total growth) is quantified in our model by the parameter *k*, which measures the slope of changes in the current salinity optimum with respect to the acclimation salinity (dashed line in Fig. 1A.B). We found a positive value of *k* (0.67, Table 1), consistent with the beneficial acclimation hypothesis (Bennett and Lenski 2007; Leroi et al. 1994), whereby higher acclimation salinity displaces the current optimum toward higher salinity. Above *S*_0_ = 1.5 M, acclimation salinity had a much lower impact on fitness response to current salinity (background color in Fig. 1A).

Acclimation's impact on total growth rate partly resulted from its impact on cell death. In addition to lower apparent growth rates (Fig. 1B), acclimation at low salinity (*S*_0_ ≤ 1.5 M) led to a large increase in the apparent instantaneous mortality, with up to 50% death at high salinities (Fig. 1C). Negative apparent growth was estimated under some conditions, implying that in addition to the deaths we could detect, other dead cells disappeared before cytometric measures. This could be caused by rapid degradation of cell structure, as occurs notably under programmed cell death, which is known to occur in *D. salina* as well as other phytoplankton (Bidle 2016; Orellana et al. 2013). In addition to its beneficial effect on growth, acclimation at high salinity essentially abolished mortality at high assay salinity (Fig. 1B and negative interaction term on mortality δ_01_ , Table 1), contributing to increasing the growth rate.

**Figure 2:**
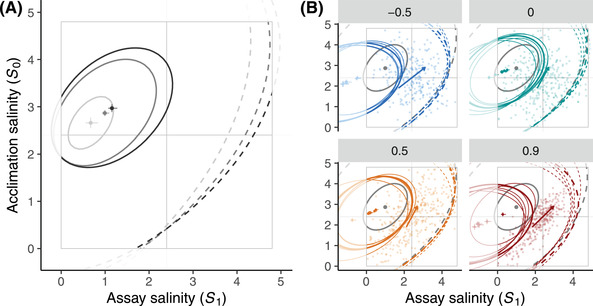
Experimental evolution of the acclimated tolerance surfaces. (A) Estimated acclimated tolerance surfaces for populations that evolved under constant salinities (from light gray to black: 0.8, 2.4, and 3.2 M). (B) Estimated acclimated tolerance surfaces for population that evolved in fluctuating salinities (thin colored lines) and prediction (thick colored line) for a population that evolved under stationary mean 2.4 M, variance 1, and autocorrelations −0.22, 0.05, 0.53, and 0.78 (from blue to red), corresponding to the mean realized autocorrelation experienced by the four or five populations under each fluctuating treatment (−0.5, 0, 0.5, and 0.9). The constant treatment with salinity 2.4 M (same as the mean of fluctuating treatments) is also shown in gray. Thick colored lines represent two isoclines of the fitness surface predicted by equations (3) to (5), for r=0 (dashed) andr= 0.85 per day (solid lines). Dots and error bars represent the estimates and standard errors for the salinity optimum, that is, the pair of acclimation and assay salinities maximizing fitness. Arrows represent the strength of acclimation, with steeper slope 1/*k* indicating weaker acclimation effects, whereby the optimal assay salinity is less dependent on the acclimation salinity.

By plotting growth rate against past and current environment, the acclimated tolerance surface can be compared to the bivariate distribution of acclimation and assay salinities, the orientation of which is controlled by temporal autocorrelation (background dots in Fig. 2B). If environmental patterns encountered more frequently exert stronger net selection, we can hypothesize that tolerance surfaces (and in particular acclimation *k*) should evolve to be aligned with the bivariate distribution of past and current salinities.

### EVOLUTION OF ACCLIMATED TOLERANCE SURFACES

After about 200 generations of experimental evolution under constant or fluctuating salinity, the shapes of acclimated tolerance surfaces evolved in response to our environmental treatments. The effects of environmental mean, variance, autocorrelation, and predictability on each of the tolerance surface parameters are summarized in Table 2.

**Table 2:** Impact of environmental moments of the salinity time series on evolution of the acclimated tolerance curve

		Intercept	μ_S_	$\sigma_{S}^{2}$	ρ_S_	$\rho_{S}^{2}$
*r*_max_	Estimate	0.949	0.043	**0.193**	–0.198	0.378
	Standard Error	0.019	0.020	**0.030**	0.095	0.136
	*P*‐value	0.00 × 10^0^	3.44 × 10^–2^	**7.05 × 10^–11^**	3.84 × 10^–2^	5.55 × 10^–3^
μ_0_	Estimate	2.865	0.129	–0.243	**1.393**	**–2.749**
	Standard Error	0.063	0.061	0.086	**0.286**	**0.449**
	*P*‐value	0.00 × 10^0^	3.38 × 10^–2^	4.76 × 10^–3^	**1.09 × 10^–6^**	**9.53 × 10^–10^**
μ_1_	Estimate	0.993	0.201	**–0.933**	**3.747**	**–6.658**
	Standard Error	0.099	0.086	**0.150**	**0.586**	**0.841**
	*P*‐value	1.33 × 10^–23^	1.95 × 10^–2^	**5.10 × 10^–10^**	**1.66 × 10^–10^**	**2.48 × 10^–15^**
*k*	Estimate	0.414	0.040	**0.185**	**–0.831**	**1.573**
	Standard Error	0.030	0.026	**0.043**	**0.132**	**0.192**
	*P*‐value	2.59 × 10^–44^	1.17 × 10^–1^	**1.46 × 10^–5^**	**3.45 × 10^–10^**	**2.43 × 10^–16^**
$1/\sigma_{0}^{2}$	Estimate	0.086	0.009	0.015	–0.004	0.012
	Standard Error	0.005	0.005	0.007	0.016	0.024
	*P*‐value	1.54 × 10^–68^	8.29 × 10^–2^	1.95 × 10^–2^	8.23 × 10^–1^	6.15 × 10^–1^
$1/\sigma_{1}^{2}$	Estimate	0.080	–0.002	**–0.020**	**0.056**	**–0.094**
	Standard Error	0.004	0.003	**0.003**	**0.007**	**0.010**
	*P*‐value	5.43 × 10^–99^	5.83 × 10^–1^	**2.28 × 10^–10^**	**7.27 × 10^–16^**	**3.13 × 10^–21^**
δ	Estimate	–3.774	0.003	0.058	0.823	–1.349
	Standard Error	0.135	0.128	0.169	0.452	0.687
	*P*‐value	3.43 × 10^–173^	9.79 × 10^–1^	7.29 × 10^–1^	6.87 × 10^–2^	4.95 × 10^–2^
δ_0_	Estimate	–0.009	–0.143	0.002	–0.199	0.212
	Standard Error	0.061	0.053	0.079	0.201	0.309
	*P*‐value	8.80 × 10^–1^	6.85 × 10^–3^	9.80 × 10^–1^	3.23 × 10^–1^	4.92 × 10^–1^
δ_1_	Estimate	0.946	0.045	0.110	–0.377	0.587
	Standard Error	0.048	0.047	0.060	0.159	0.241
	*P*‐value	2.24 × 10^–87^	3.42 × 10^–1^	6.92 × 10^–2^	1.75 × 10^–2^	1.48 × 10^–2^
δ_01_	Estimate	0.946	0.045	0.110	–0.377	0.587
	Standard Error	0.048	0.047	0.060	0.159	0.241
	*P*‐value	2.24 × 10^–87^	3.42 × 10^–1^	6.92 × 10^–2^	1.75 × 10^–2^	1.48 × 10^–2^

*Note*: Each row corresponds to one parameter of the bivariate function relating growth or mortality to current and previous salinity (eqs. 3 and 4). Each column gives the estimate, standard error, and *P*‐value from Wald test for the effect of each salinity moment (mean $\overset{-}{S}$variance $\sigma_{S}^{2}$, autocorrelation ρ_S_, and predictability $\rho_{S}^{2}$). Effects of the evolutionary treatment that are significant after Bonferroni correction for multiple (40) comparisons appear in bold.

#### Evolutionary niche shift in response to the mean salinity

The mean salinity experienced during evolution had a highly significant effect on acclimated tolerance surfaces (*P* < 2 × 10^–16^, likelihood‐ratio test—hereafter LRT—between models with vs. without effects of salinity mean). The mean environment jointly affected all parameters, but without strong shift in any specific parameter (Table 2). However, there was weak evidence that higher mean salinity during evolution increased the optimum with respect to current $(\mu_{0})\;$and past $(\mu_{1})\;$salinities (Table 2), and increased the protective effect of higher acclimation salinity on the apparent death ratio (Table 2, δ_0_). Such effects appeared clearly when considering only the constant treatments, for which the optimum salinity shifted toward higher values on both axes as the evolution salinity increases (dots and error bars in Fig. 2B). In addition the niche breadth, that is, the range of salinities leading to a positive growth rate (dashed isocline in Fig. 2B) and the salinity range with high growth rates (>0.85, solid isoclines in Fig. 2B) were both expanded toward higher assay salinities for populations that evolved at 2.4 and 3.2 M as compared to 0.8 M.

When assessed in specific cross‐salinity transfers (acclimation *S*_0_ × assay *S*_1_), growth rates of populations that evolved at higher salinities were significantly higher for transfers to extremely high salinities (4.3 and 4.7 M), and for transfer from near‐freshwater (0.1 M) to 1.8 M (Table S1). In contrast, none of the salinity transfers from acclimation salinities *S*_0_ > 1.5 M showed evolutionary difference in growth rates between constant treatments. Hence, evolutionary changes in response to the mean salinity mostly involved transfers from low to medium/high salinity.

#### Environmental fluctuations led to increased tolerance breadth

The magnitude of salinity fluctuations during evolution significantly impacted the shape of the acclimated tolerance surface (LRT between the full model and a model without effect of variance in salinity: *P*‐value < 2 × 10^–16^). In particular, populations that evolved in randomly fluctuating environments had a significantly broader tolerance curve with respect to the current salinity (lower $1/\sigma_{1}^{2}$; Table 2; Fig. 2B), and their optimum assay salinity μ_1_ shifted toward lower values (Fig. 2B), whereas their maximal growth rate *r*_max_ increased by about 20% (Fig. 2B).

We did not find any significant effect of environmental variance on the mortality surface. However, when analyzing responses more finely at the level of each salinity transfer, we found that the death ratio significantly increased with increasing salinity variance for transfers from 1.1 to 3, 3.7, and 4.3 M (Bonferroni correction for multiple testing; see Table S2), even though the net growth rate in fact increased with salinity variance for the latter transfer.

#### Increased acclimation effects evolved in more predictable environments

We found a significant impact of environmental autocorrelation *ρ* and predictability *ρ* ^2^ during evolution on the shape of the tolerance surface (*P* = 5.4 × 10^–15^/*P* = 6.5 × 10^–8^, LRT between the full model and the model without autocorrelation/predictability). Overall, very similar tolerance curves were found for treatments 0 and 0.5 (green and orange lines in Fig. 2B), which differed from the group pairing treatments −0.5 and 0.9 (blue and red lines). The acclimation slope (*k*, Table 2), which measures how the optimum with respect to current salinity depends on the previous salinity, was higher in populations that evolved in more predictable environments, which also had higher tolerance breadth (larger σ_1_). In addition, optimal salinities in the acclimation and assay phases both shifted toward lower values with increased predictability.

### GLYCEROL PLASTICITY EVOLVED IN RESPONSE TO ENVIRONMENTAL PREDICTABILITY

**Figure 3:**
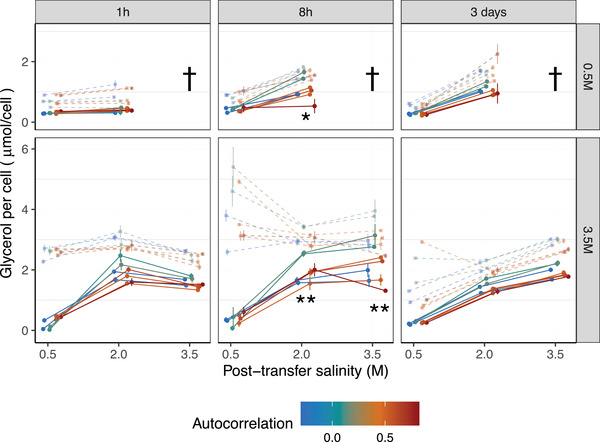
Evolution of plasticity in glycerol content. The reaction norms of intracellular (darker symbols, continuous lines) and total (lighter symbols, dashed lines) glycerol concentration per *Dunaliella* cell against assay salinity are represented for different autocorrelations during experimental evolution (colors). Measurements made 1 hour, 8 hours, and 3 days after a salinity change are displayed in different columns, whereas the rows correspond to different acclimation salinities. Stars indicate a significant joint effect of autocorrelation and predictability treated as continuous variables (*P < 0.05 or **P < 0.01 after Bonferroni correction) on intracellular glycerol content in the corresponding conditions (time and cross‐salinity treatment).

We investigated how the level of glycerol, an osmoprotectant known to be the main mechanism of salinity tolerance in *D. salina* (Ginzburg 1988), evolved in response to our experimental evolution treatments. Intracellular glycerol concentration responded plastically to salinity, increasing in transfers from low to high salinity, and decreasing for transfers from high to low salinities, consistent with previous findings in this species (Ben‐Amotz and Avron 1973). The dynamics of glycerol content was asymmetric (as also shown in Rescan et al. 2020): glycerol concentration dropped faster after a transfer from 3.5 to 2 M (bottom left in Fig. 3) than it increased after a transfer from 0.5 to 2 M (middle points and curve in top‐left panel in Fig. 3), because the former may involve rapid excretion (Zidan et al. 1987).

We detected evolution of plasticity of intracellular glycerol content in response to our predictability treatments. A significant interaction between assay salinity *S*_1_ (treated as a categorical variable) and environmental predictability ρ^2^ (*P*‐value = 2.82 × 10^–3^, ANOVA on 1000 imputed dataset; Table S4) demonstrated that lines that experienced different predictabilities during experimental evolution responded differently to their current salinity. Environmental predictability and autocorrelation also affected the baseline glycerol content, regardless of plasticity (*P*‐value = 1.87 × 10^–2^ and 6.69 × 10^–3^ for *ρ* ^2^ and *ρ*; Table S4). These parameters of the evolutionary treatments (*ρ*, *ρ* ^2^ and interaction *ρ* ^2^:*S*_1_) jointly explained 25% of the phenotypic variance that remained, after accounting for the effects of acclimation and assay salinities, and the timing of glycerol measurement. The overall magnitude of plasticity, regardless of reaction norm shape, can be quantified by the among‐environment component of phenotypic variance. Here, we found a negative effect of *ρ* ^2^ on the plastic variance in glycerol content among salinities (−7 × 10^–11^, *P*‐value = 4.1 × 10^–2^, multiple regression on the variance estimated for the 1000 imputed datasets), indicating that lower plasticity levels evolved in populations exposed to more predictable environments, mostly because of a weaker response to the assay salinity (Fig. S5). This translates the fact that populations from less predictable environments had higher intracellular glycerol levels than other evolutionary treatments at high salinities (2 and 3.5 M), but similarly low level at 0.5 M (green vs. other colors in Fig. 3; Table S3). This pattern is most marked 8 hours posttransfer, when moving from 0.5 to 2 M, and from 3.5 to 2 M or to 3.5 M (Table S3). Note that evolution of plasticity was only weakly significant when assay salinity was instead treated as a continuous variable (*P*‐value = 3.97 × 10^–2^ for the interaction between *S*_1_ and *ρ* ^2^, ANOVA on 1000 imputed dataset), indicating that evolutionary changes in plasticity were poorly captured by changes in the slope of a linear reaction norm.

Environmental predictability also affected the plasticity of extracellular glycerol content, as evidence by a significant interaction between acclimation salinity *S*_0_ and environmental predictability and autocorrelation (*P*‐value = 8.1 × 10^–3^ and 2.2 × 10^–2^, respectively, mi.anova on 1000 imputed dataset; Table S5). The variance of intracellular glycerol across assay environments (a measure of the overall magnitude of plasticity) decreased with increasing predictability of the evolutionary treatment (−2.4 × 10^–10^, *P*‐value = 7.9 × 10^–3^, multiple regression on the variance estimated for the 1000 imputed datasets), implying that extracellular glycerol was also more plastic in populations that evolved in less predictable environments. In addition, baseline extracellular glycerol level was impacted by the variance of environmental fluctuations (Table S5; *P*‐value = 2.0 × 10^–3^, AVONA on 1000 imputed dataset), and significantly increased with higher salinity variance during evolution, both 8 hours (*P* = 1.43 × 10^–5^) and 3 days (*P* = 4.25 × 10^–6^) after a transfer from 3.5 to 0.5 M. Since extracellular glycerol necessarily originates from *Dunaliella* in our experiment, this suggests that after a salinity drop, more glycerol was not only produced, but also excreted (as documented in this species; Zidan et al. 1987), in populations having experienced larger fluctuations during experimental evolution.

## Discussion

We exposed lines of the halotolerant microalga *D. salina* to constant and randomly fluctuating salinity for several months, to investigate how patterns of environmental fluctuations, especially their predictability, influenced the evolution of its fundamental niche with respect to salinity. By measuring population growth in 75 alternative combinations of acclimation × assay salinities, we were able to estimate a bivariate tolerance surface with respect to past and current environments. This allowed us to show that the acclimation salinity during days prior to the assay had a major impact on population growth in *Dunaliella*. In particular, we found a strong decrease in growth, and increase in apparent mortality, following a transfer from low to high salinities (Fig. 1).

### EVOLUTION OF TOLERANCE SURFACES

Beyond these general characteristics of the acclimated tolerance surface, we were able to analyze its evolution along its two dimensions. First, we evidenced adaptation to the mean environment, with higher mean salinity during evolution leading to an increase in the optimum, not only with respect to current, but also to acclimation salinity. Second, populations that evolved under fluctuating salinity had broader tolerance than those from constant treatments. Third and more originally, we found that the predictability of environmental fluctuations (as measured by their squared autocorrelation) also had a number of evolutionary impacts on the tolerance surface. In particular, more predictable environments led to stronger influence of the acclimation environment on the optimal assay environments (as measured by the slope *k*), implying that current salinity tolerance was more conditioned by past salinity. Higher environmental predictability also led to lower optimal salinity (for acclimation and assay), and to broader tolerance curves (with respect to the assay salinity). Note, however, that caution is warranted when interpreting the breadth parameter of the tolerance surface directly in terms of niche limits. Indeed, salinity cannot be negative, whereas the maximum salinity allowing positive growth depends not only on the breadth, but also on the position and height of the tolerance surface. For instance, the populations that evolve at ρ=0 have the smallest tolerance breadth, but their salinity niche is still larger because they have a higher optimum while still being able to grow at the minimum salinity (Fig. 2B).

### THE ROLE OF GLYCEROL PLASTICITY

To gain more mechanistic insights on these evolutionary responses, we analyzed the dynamics of glycerol content, a compatible solute used as osmoprotectant by *D. salina* (Chen and Jiang 2009). The asymmetric dynamics of glycerol content, with fast decline under reduced salinity (notably via excretion) but slow rise under increased salinity (Fig. 3), likely underlies asymmetries in the tolerance surface (Fig. 1), as previously highlighted in Rescan et al. (2020). Regarding evolutionary differences among treatments, the glycerol dynamics in Figure 3 helps shed light on why salinity optima in the tolerance surface increased with environmental predictability. After transfers to salinities above 0.5 M, populations that evolved in less predictable environments had higher intracellular glycerol level. Intracellular glycerol concentration even increased without any salinity change (compare 1 vs. 8 hours in transfer from 3.5 to 3.5 M in Fig. 3), before decreasing back toward its initial value after 3 days. Unpredictable environments were therefore associated with an ability to produce and maintain higher levels of intracellular glycerol at intermediate to high salinities. This may explain the increase in (current) salinity optimum in populations from unpredictable environments. Beyond its osmoregulatory effect, higher glycerol level may also provide an energetic source, as suggested by higher growth rates at low salinities of populations coming from high to medium salinity (and thus containing high levels of glycerol), as compared to those that acclimated at low salinity.

The combination of low glycerol content at low salinity and high glycerol content at medium/high salinity translated to evolution of higher plasticity of intracellular glycerol in populations that evolved in less predictable environments. Although this may seem to contradict the narrower tolerance breadth of these populations, it is actually consistent with their higher upper salinity niche limit (Fig. 2B, upper right). We also found higher plasticity of extracellular glycerol in response to past salinities in lines that evolved in less predictable environments, and higher baseline extracellular glycerol content in populations that evolved in more variable environments, suggesting higher glycerol excretion by *D. salina*. This trait may play an important role in its natural environment, where this species co‐occurs with heterotrophic bacteria (e.g., *Halobacterium salinarum*) and archaea (mainly *Haloquadratum*, and Halobacteriaceae such as *Halorubrum*), which are able to remineralize organic carbon released by phytoplankton, thus contributing to the so‐called microbial‐loop in the aquatic carbon cycle (Baines and Pace 1991).

### LIMITED CHANGE IN TOLERANCE SURFACES

Overall, the changes in tolerance surfaces that we detected remained subtle, even after 200 generations of evolution. *Dunaliella salina* conserved the broad salinity niche characteristic of a halotolerant species, even after more than 200 generations in approximately seawater salinity (for the constant low salinity treatment), and evolution of optimum salinity did not lead to reduced growth at low salinity (no detected trade‐off). The general shape was also conserved across predictability treatments (Fig. S2), rather than rotating to align with the pattern of environmental fluctuations (light dots in Fig. 2B), which suggests that salinity tolerance surfaces are quite constrained in *Dunaliella* (Zhao et al. 2013). Nevertheless, our detailed measurement of tolerance surfaces across a broad combination of acclimation and assay salinities still allowed us to detect evolutionary responses that would probably have gone unnoticed using simpler approaches. Partitioning of apparent death and growth rates confirmed that in phytoplankton, mortality becomes proportional to the abiotic stress when it exceeds the upper tolerance limit (as found with thermal stress in a diatom; Baker and Geider 2021). On the other hand, we were constrained to use a rather crude population growth model, assuming exponential growth to estimate a net per capita growth rate over 3 days. During this time lapse, populations might have experienced more complex population dynamics, including lags preceding exponential growth, or even decline followed by population rebound (Leung et al. 2020; Zeballos et al. unplubl. ms.). Therefore, larger evolutionary responses of tolerance surfaces may have been revealed by a more detailed analysis of the population dynamics to include these effects. This is especially expected if some growth phases are more sensitive than others to the environment, as shown previously for cell morphology (Leung et al. 2020).

### RELATION TO THEORETICAL PREDICTIONS

How do our results relate to theoretical predictions about evolution of environmental tolerance, and the plasticity of underlying traits? The larger tolerance breadth that evolved in population exposed to larger fluctuations is associated with a higher maximal growth rate, contrary to expectations under a generalist‐specialist trade‐off caused by a cost of plasticity (Chevin et al. 2010; Lynch and Gabriel 1987; Van Tienderen 1991), but consistent with findings in other experimental systems (Karve et al. 2016; Kassen 2002; Scheiner 2002). The mechanisms involved in salinity tolerance may not be very costly in our system, given that niche breadth remains broad after evolution in constant salinities. Here, the main trait involved in osmotic resistance is intracellular glycerol content, which adjusts continuously to the salinity (Ben‐Amotz and Avron 1973). Theoretical models predict that the level of plasticity at evolutionary equilibrium should be proportional to environmental autocorrelation, for a trait that is fixed during development prior to selection (Gavrilets and Scheiner 1993; De Jong 1999; Lande 2009; Levins 1963; Moran 1992), whereas for a labile trait, this is only true if there is a cost of plasticity (Lande 2014). Our result that higher intracellular glycerol plasticity evolved in populations exposed to unpredictable environments does not conform to these predictions, and surprisingly contrasts with previous results on the evolution morphological plasticity in the same experiment (Leung et al. 2020). The pattern we found here likely reflects that populations from unpredictable environments consistently produced higher glycerol at medium to high salinity, but not at low salinity (0.5 M). This response may be adaptive, because the severe mortality induced by transitions from low to high salinity results in an asymmetric fitness function. Theory has shown that such asymmetry can promote the evolution of excessive phenotypes, such as overly costly immune defenses, all the more as phenotypic variance is large (Urban et al. 2013). Here, the cost of delaying glycerol excretion at low salinity is likely to be much lower than the benefit of retaining high glycerol content in high salinities, such that it may be beneficial to maintain hyper‐optimal glycerol levels when cues about future salinity are unreliable. More generally, the observation that glycerol plasticity did evolve in response to environmental predictability, but in a direction opposite to most theoretical predictions, suggests that these predictions may be challenged when the simplifying assumptions they rely on (e.g., linear reaction norms, symmetric fitness functions) are violated, and calls for novel theory exploring these effects.

### PREDICTABILITY VERSUS AUTOCORRELATION

Another puzzling result was that similar tolerance surfaces evolved in negatively and highly positively autocorrelated environments. This is all the more surprising as a strong acclimation effect should be detrimental under negative autocorrelation. Although surprising, this result is consistent with previous observations from the same experiment, where both the evolution of morphological plasticity (Leung et al. 2020) and the direction and strength of selection among competing strains (Rescan et al. 2021) depended not on the environmental autocorrelation, but rather on its square (environmental predictability), such that negative and positive autocorrelations with similar magnitudes produced similar evolutionary outcomes. Possible explanations may involve an effect of the salinity two transfers in the past, with correlation ρ^2^ to the current salinity. In fast growing microbes such as *D. salina*, daughter cells inherit large portions of their cytoplasm from their progenitors, such that with salinity changes every three or four generations in average, salinity two transfers before may impact current intracellular content, fitness, and therefore selection. These somewhat unexpected results highlight how confronting the outcome of experimental evolution with theoretical predictions can yield interesting insights, and open possible avenues for further theory. In particular, most theory on the evolution of plasticity does not address the dynamics of plastic changes and tolerance curves (but see Gabriel et al. 2005; Lande 2014), including asymmetries in rates of responses, and current consequences of past changes. Incorporating these processes more explicitly may lead to substantial theoretical progress and stronger connection with empirical results.

## AUTHOR CONTRIBUTIONS

L‐MC and MR designed the experiment. MR and DG performed the experimental evolution experiment and MR, DG and NL performed the tolerance curve and glycerol dynamics experiment. MR analysed the data. MR and L‐MC wrote the original draft. All authors reviewed and approved the final draft of the paper.

## CONFLICT OF INTEREST

The authors declare no conflict of interest.

## Supporting information

Supplementary Figures

**Supplementary Table 1**: Effect of constant salinity on total growth, apparent growth and apparent death ratio, in all acclimation x assay salinities.
**Supplementary Table 2**: Influence of evolutionary treatment (salinity mean, variance, autocorrelation and predictability) on total growth, apparent growth, and apparent death ratio in all acclimation x assay salinities.
**Supplementary Table 3**: Linear regressions of intracellular, extracellular and total glycerol content (scaled per cell) on the parameters of the evolutionary treatment (mean, variance, autocorrelation and predictability).
**Supplementary Table 4**: Partitioning of variation in intracellular glycerol content.
**Supplementary Table 5**: Partitioning of variation in extracellular glycerol content.

## Figures and Tables

**Figure A1:**
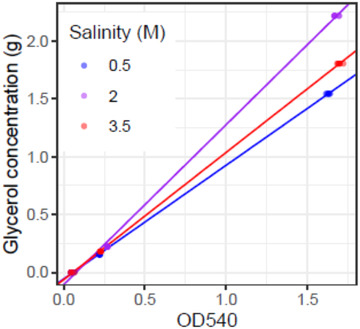
Glycerol measure calibration

## Appendix A Standard Glycerol Calibration

Glycerol concentrations extrapolation from optic density at 540 nm requires a standard calibration in conditions similar to those of the experimental measures. We prepared 0, 1.5, and 15 mM glycerol solutions with salinities 0.5, 2, and 3 M. Exact weights were measured to account for dilution errors induced by glycerol high viscosity. Optical density at 540 nm (OD540) was measured after adding 800 μL glycerol reagent solution to 200 μL of each of the standard glycerol solution. Three replicates were measured for each salinity and standard glycerol concentration, and three additional replicates were taken for each condition after glycerol solution filtration (2000 rpm, 5 minutes).

We regressed glycerol concentration against OD540 (Table A1). As expected, the filtration procedure did not impact the regression between glycerol concentration and OD540. However, we found significant differences between salinities in both the intercept and the slope of the regression. We therefore performed a linear regression between glycerol concentration for each salinity (Table A2; Fig. A1).

**Table A1:** Effect of filtration and salinities on glycerol measure calibration

	Estimate	Standard Error	Pr(>|*t*|)
Intercept	−0.059516	0.00868	1.49 × 10^–8^
Salinity[2]	−0.049789	0.010675	2.69 × 10^–5^
Salinity[3.5]	−0.006131	0.010595	0.566
Filtration	0.006062	0.008689	0.489
OD540	0.983862	0.00909	<2 × 10^–16^
OD540:Salinity[2]	0.400285	0.011062	<2 × 10^–16^
OD540:Salinity[3.5]	0.117126	0.010936	4.39 × 10^–14^
OD540:Filtration	−0.003897	0.008926	0.664

**Table A2:** Regression between glycerol concentration and OD540 at the three salinities used in the experiment

Salinity	Intercept	Slope
0.5	−0.056469	0.981893
2	−0.106270	1.382195
3.5	−0.062603	1.099026
