# Supplementary material for: Experimental evolution of environmental tolerance, acclimation, and physiological plasticity in a randomly fluctuating environment
Source: Evol Lett. 2022 Dec 7;6(6):522–36. doi: 10.1002/evl3.306 (PMC9783450; doi:10.1002/evl3.306)
Supplement: Supplementary file 1 — Supplementary Figures [file EVL3-6-522-s001.docx]

# Supplementary Figures


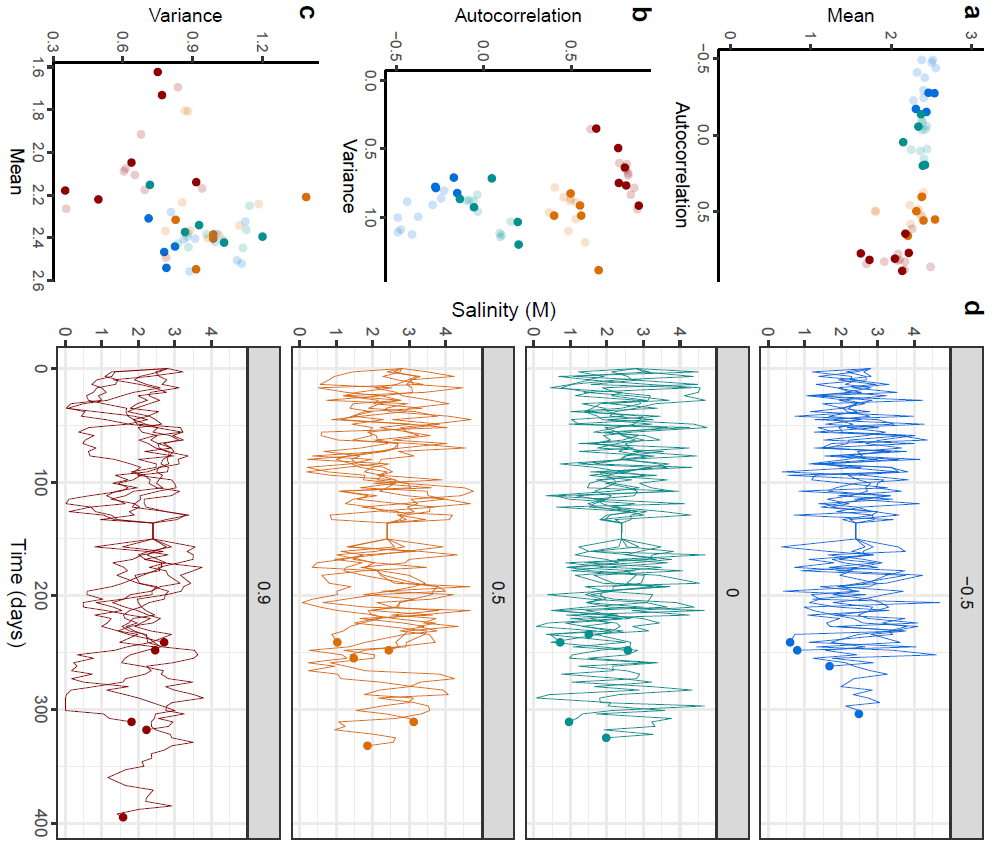
**Supplementary Figure 1: Salinity time series and realized moments of experimental evolution treatments**. The relationships between the moments (mean, variance, and autocorrelation) of the salinity times series are represented in (a)-(c), while the full stochastic salinity time series appear in (d). Colors represent the target, stationary value of the autocorrelation treatment: -0.5 (blue), 0 (green), 0.5 (orange), 0.9 (red). In a, b and c, darker dots correspond to the time series (shown in d) experienced by the 19 populations analyzed in this study. Lighter dots represent the 26 populations evolving under the same stochastic processes but that have not been used in this analysis, as part of them got extinct before the end of the experiment.

**Supplementary Figure 2: Acclimated tolerance curves of the 25 populations evolved in stochastic or constant salinities**. Each panel represents one population. Background color gives the growth rate estimated for each salinity transfer (*S*_0_ to *S*_1_) and contour lines are isoclines of the growth and the death function estimated by Eqs.2, 3 and 4.


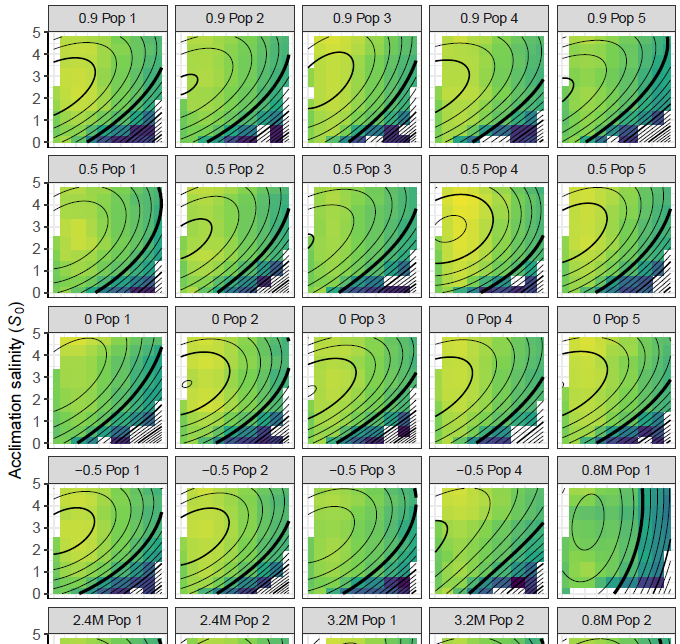

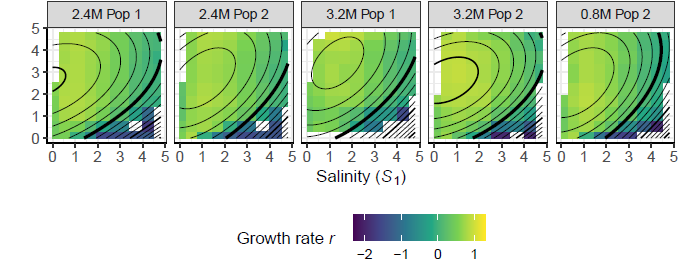


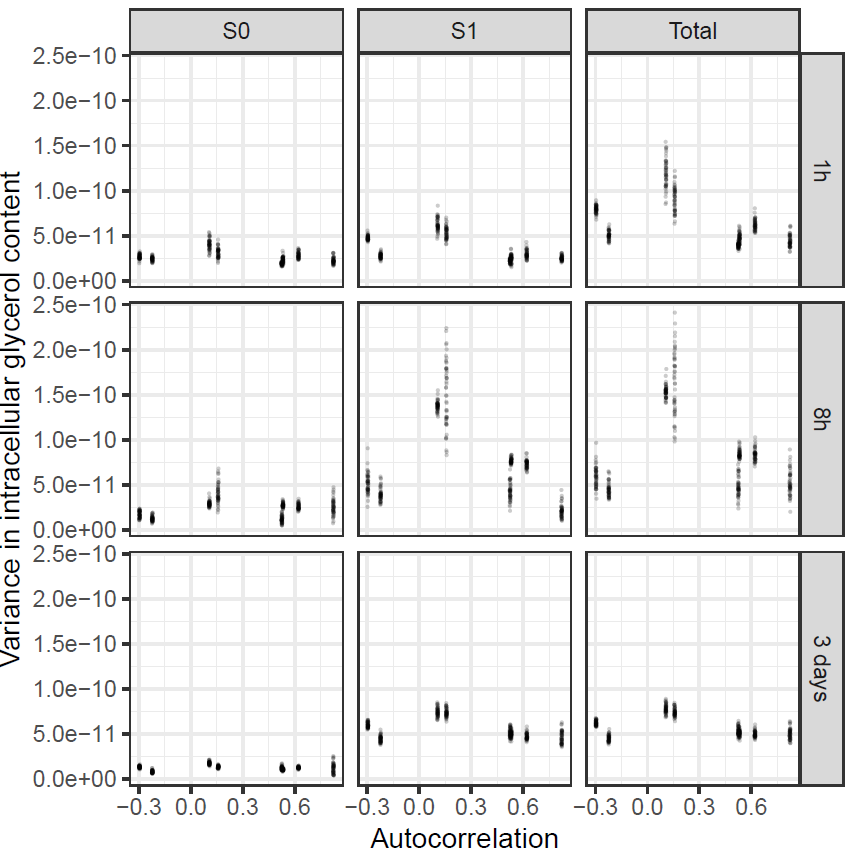


**Supplementary Figure 3: Autocorrelation effect on the variance in intracellular glycerol due to phenotypic plasticity**. The total variance of environmental effects on glycerol content, and the variances linked to past (S_0_) and current (S_1_) salinities (columns), all increased with decreasing environmental predictability (square of temporal autocorrelation, x-axis). For each point, variance in intracellular glycerol content across the 6 salinity transfers was computed for 1000 datasets, where glycerol contents were sampled from a normal distribution with parameters (mean, error) estimated from our empirical measurements.
